# Validation of a Cochlear Implant Patient-Specific Model of the Voltage Distribution in a Clinical Setting

**DOI:** 10.3389/fbioe.2016.00084

**Published:** 2016-11-23

**Authors:** Waldo Nogueira, Daniel Schurzig, Andreas Büchner, Richard T. Penninger, Waldemar Würfel

**Affiliations:** ^1^Department of Otolaryngology, Cluster of Excellence “Hearing4all”, Medical University Hannover, Hannover, Germany

**Keywords:** cochlear implant, finite element model, cochlea anatomy, voltage distribution, impedance measure

## Abstract

Cochlear Implants (CIs) are medical implantable devices that can restore the sense of hearing in people with profound hearing loss. Clinical trials assessing speech intelligibility in CI users have found large intersubject variability. One possibility to explain the variability is the individual differences in the interface created between electrodes of the CI and the auditory nerve. In order to understand the variability, models of the voltage distribution of the electrically stimulated cochlea may be useful. With this purpose in mind, we developed a parametric model that can be adapted to each CI user based on landmarks from individual cone beam computed tomography (CBCT) scans of the cochlea before and after implantation. The conductivity values of each cochlea compartment as well as the weighting factors of different grounding modes have also been parameterized. Simulations were performed modeling the cochlea and electrode positions of 12 CI users. Three models were compared with different levels of detail: a homogeneous model (HM), a non-patient-specific model (NPSM), and a patient-specific model (PSM). The model simulations were compared with voltage distribution measurements obtained from the backward telemetry of the 12 CI users. Results show that the PSM produces the lowest error when predicting individual voltage distributions. Given a patient-specific geometry and electrode positions, we show an example on how to optimize the parameters of the model and how to couple it to an auditory nerve model. The model here presented may help to understand speech performance variability and support the development of new sound coding strategies for CIs.

## Introduction

Cochlear implants (CIs) are implantable medical devices that are used to restore the sense of hearing for people with profound hearing loss or deafness given that the auditory anatomy is fully developed (Wilson et al., 1991). Cochlear implantation (and subsequent rehabilitation) typically allows even children with prelingual deafness to develop spoken language understanding and production (De Raeve, 2010; Colletti et al., 2012).

In Cis, the auditory nerve fibers are directly stimulated using an array of electrodes, bypassing the natural functioning of the outer, middle, and inner ear. Current is applied to an electrode to directly elicit action potentials in the auditory nerve. In a natural cochlea, a pure tone produces neural excitation at a specific region corresponding to an auditory filter with a relatively narrow bandwidth (Greenwood, 1990). In CIs however, a broad excitation is produced, mainly because the fluids in the cochlea are highly conductive causing the charge to spread along the inner ear. This phenomenon is commonly referred to as spread of excitation. The amount of spiral ganglion cell survival and the degree of dendrite degeneration might also contribute to sound perception. For example, if a narrower field is applied to a region of the cochlea with a low number of active neurons, the current will have to be increased to reach neighboring neurons, and the spread of excitation will automatically become wider. It has been shown that the number of independent spectral channels in the signal determines intelligibility (Shannon et al., 2004), so that spread of excitation should reduce intelligibility to the extent that it reduces the number of channels. The spread of excitation is influenced by the anatomy and the conductivity of the tissues in the cochlea (Finley et al., 1990; Frijns et al., 1995; Briaire, 2008; Saba, 2012). This was confirmed using *in vivo* measurements of the cochlea with an implanted electrode, using resistive models and solving analytical equations of the three-dimensional (3D) volume conduction problem (Suesserman and Spelman, 1993). It is also known that the anatomy (Würfel et al., 2014) and electrode positions (Landsberger et al., 2015) differ substantially from CI user to CI user.

New sound coding strategies use multiple electrode stimulation to perform electric field shaping (e.g., Litvak et al., 2007; Landsberger et al., 2012; Nogueira et al., 2015a). However, multiple electrode CI coding strategies require precise knowledge of the voltage distribution and current flow within an electrically stimulated cochlea (Kalkman et al., 2014). 3D voltage distribution models can be applied to characterize the electrically stimulated cochlea and can therefore be a useful tool to explain some of the speech performance variability and to optimize sound coding strategies.

Three-dimensional models of the voltage distribution in the cochlea have been developed extensively in the past. The history of development of 3D cochlear models can be found in Hanekom and Hanekom (2016) and Kalkman et al. (2016). Impedance networks were used as transmission line models to calculate the voltage in the scala tympani as a function of distance from the cochlear base (Suesserman and Spelman, 1993). It was reported that these types of models are of value in the estimation of current interactions, but they do not provide with the resolution necessary to simulate its individual excitation process. In order to obtain more accurate simulations, the employment of the boundary element method (BEM) and finite element method (FEM) were proposed. Finley et al. (1990) were the first to present an integrated 3D neuron field model of a segment of an unrolled cochlea using the FEM. Frijns et al. (1995, 1996) presented a rotationally symmetric cochlear geometry for the calculation of neural excitation patterns using different electrode configurations and stimulation patterns. In Briaire (2008), the model was improved using a more refined helical representation of the cochlea. Kalkman et al. (2014) extended the BEM model based on a spiral shaped cochlea to simulate spread of excitation with simultaneous electrode stimulation. In Rattay et al. (2001), a simplified spiraled model of the human cochlea was developed from a cross-sectional microphotography as well. More recently a 3D FEM model of the cochlea was developed to obtain the voltage distribution at positions closer to the site of neural stimulation (Nogueira et al., 2015b). This model was used to demonstrate the way the voltage distribution varies with the geometry of the cochlea and the electrode array.

Most FEM CI models have not been developed parametrically such that they can be adapted to each CI user in a flexible manner. If the parameterization is available, it can be adapted to each CI user from clinical data for optimization. In the work of Whiten (2007), two donor cochleae were individualized post-mortem using high-resolution CT. The individualized models were then used to predict measures obtained *in vivo*. Other models (Suesserman and Spelman, 1993; Hanekom, 2001; Briaire, 2008) are very sophisticated trying to model many details of the cochlea; however, their adaptation to each CI user seems to be time consuming. Recently, geometrical models have been developed such that they can be adapted to each CI user (Dang et al., 2015; Malherbe et al., 2015b; Mangado et al., 2016). In Dang et al. (2015), a detailed anatomical model of the cochlea was parameterized and used to predict the voltage distribution (using FEM) created by CI electrical stimulation in a temporal bone. In Malherbe et al. (2015a,b), a method to construct user-specific models of the cochlear of living CI users was presented. In their study, the effect of variations in cochlear morphology and electrode location on modeled potential distributions and neural excitations was analyzed. They showed that the effect of morphology is almost as significant as the effect of electrode location. Therefore, a model that takes into account both, the electrode location as well as the morphology of the cochlea, is required to obtain more reliable estimations of the voltage distribution and the neural activity of each CI user. Such a model can be useful to better understand intersubject performance variability in CI users. However, these models have not been validated and compared to *in vivo* electric recordings obtained from CI users in a clinical setting.

In this manuscript, we present a simple and parametric patient-specific 3D-FEM model of the electrically stimulated cochlea. The model allows the investigation of the effects of electrical spread of excitation for different cochlear geometries adapted to individual CI users. Individualization of the model is based on clinical imaging data. The cochlear geometry can be obtained prior to cochlear implantation using clinical cone beam computed tomography (CBCT) (Würfel et al., 2014). The estimated geometry can be approximated using functions with only a few parameters (Cohen et al., 1996; Escudé et al., 2006) to create personalized 3D computer-assisted drawing (CAD) models of the cochlea. The electrode positions can be estimated after implantation using CBCT and the known dimensions of the electrode array. FEM can then be used to simulate the voltage distribution for each CAD model. Furthermore, current commercial CI systems provide a backward telemetry link to measure the voltage distribution in CI users. Such measures have been used to validate and further optimize the 3D model.

The manuscript is organized as follows: first, the methodology to build patient-specific cochlear geometries and to place the electrode positions in the model is presented. Next, a FEM model of the voltage distribution using these geometries is introduced. This modeled voltage distributions are compared to measured voltage distributions in CI users in the Section “Results.”

## Materials and Methods

This section presents the methodology to construct patient-specific models of the electrically stimulated cochlea from CBCT data. First, cochlear parameters are determined based on CBCT imaging data; second, the individualized geometry of the cochlea is constructed; third, the individualized electrode geometry is created; fourth, the electrical field is simulated using FEM; and fifth, the model is validated comparing its predictions to intra-cochlear voltage measurements in CI users.

### Cochlea Parameter Determination

Different measures of the patient’s individual cochlea and the electrode positions were extracted from preoperative and postoperative CBCT data. Preoperative scans were used to characterize the cochlea of each individual, and postoperative scans were used to assess the electrode positions inside the cochlea. Note that the postoperative scans are contaminated with artifacts produced by the metal electrodes, and this limits its use to characterize the cochlea. An aligning procedure was required to match both the preoperative and postoperative datasets because the CBCT scans were performed on different sessions and therefore, not exactly in the same position.

#### Preoperative Scan Measures

**Figure 1:**
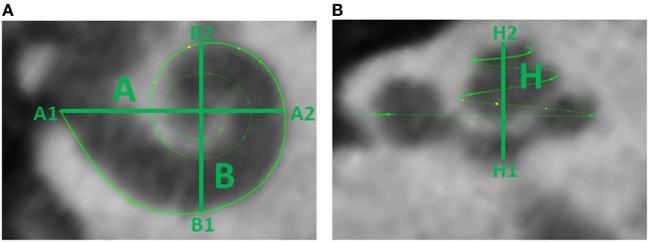
**(A)** Cochlear dimensions estimated from CBCT. *A* and *B* values in CBCT data as clinically derived measures. **(B)** Height (*H*) of the cochlea starting from the lowest basal point to the apex (Helicotrema).

Temporal bone CBCT data were collected using a stationary Xoran MiniCat (Ann Arbor, MI, USA) equipped with a 536 × 536 matrix detector resulting in 0.3 mm × 0.3 mm × 0.3 mm isotropic voxels (125 kVp, 7 mA). DICOM data processing was performed with OsiriX MD (Pixmeo, Geneva, Switzerland) using a 3D curved multiplanar reconstruction (MPR) tool. The starting point at the lateral wall of the cochlea was characterized by identifying the distal bony rim of the round window (A1 in Figure 1A). Next, a curve was set up in three-dimensions along the outer edge of the bony cochlea in projection of the osseous spiral lamina following the same method as Würfel et al. (2014). The endpoint was defined by the helicotrema (H2).

For each CI user, the *A*, *B*, and *H* values were derived from the 3D curves. The *A* value is defined as the largest distance from the round window (A1) through the modiolar axis to the lateral wall (A2). The *B* value is defined as the perpendicular distance to *A* (Escudé et al., 2006), where the geometrical points are denoted by B1 and B2. An example of these preoperative measures for a CBCT scan of a real CI user is presented in Figure 1. The *H* value is defined as the distance from the center of the modiolus in the basal turn (H1) to the helicotrema (H2).

#### Postoperative Scan Measures

**Figure 2:**
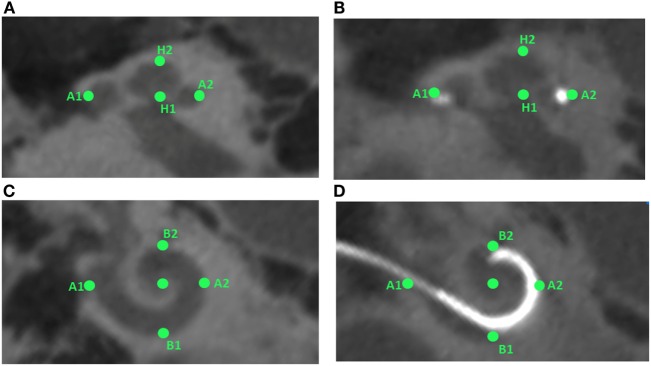
**Preoperative and postoperative scans for study participant ID1**. The left panels **(A,C)** show the preoperative scans in sagittal and axial planes, respectively, with the markers in green. The right panel **(B,D)** shows the postoperative scans in sagittal and axial planes, respectively, with markers highlighted in green color.

The same marker points (A1, A2, H1, H2, B1, and B2) were measured after implantation using the same imaging technique. Figure 2 presents the six markers measured in the same CI user in preoperative (left panel) and postoperative scans (right panel) in the sagittal (top panel) and axial planes (bottom panel).

### Individualized Geometry: Parametric Cochlear Modeling

**Figure 3:**
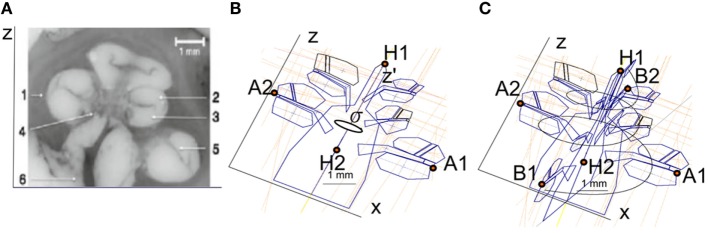
**(A)** Microphotography of a midmodiolar cross-sectional image of a human cochlea histology. 1, spiral ligament; 2, scala media; 3, scala tympani; 4, modiolus; 5, scala vestibule; 6, auditory nerve. **(B)** Compartments of a cochlea for the plane XZ. The cochlea structures are repeated every 90° around the *z*′ axis with angle σ. **(C)** Splines used to interpolate the cochlear compartments around the *z*′ axis.

The cochlear geometry was constructed following a very similar procedure as in Rattay et al. (2001). The cochlear structure was traced using Inventor© (Autodesk, Mill Valley, CA, USA). The cochlea was segmented into compartments: bone, nerve tissue, perilymph, endolymph, Reissner’s membrane, basilar membrane, and organ of Corti. In a first step, the shapes of the compartments at σ = 0°, 180°, 360°, 540°, and 720° were approximated by polygons with a relatively low number of key points (Figure 3B). Next, the same compartments were created at σ = 90°, 270°, 450°, 630°, and 810° around the *z*′ axis. The shape of the compartments was estimated using linear interpolation using the two surrounding compartments employing a similar method to the one proposed by Yoo et al. (2000). For example, the compartments at σ = 90° were obtained interpolating the compartments at σ = 0° and σ = 180°. Next, the shape of the cochlear duct and its compartments were scaled to fit the shape of two histological cross-sectional microphotographs of a human cochlea (Figure 3A).

**Figure 4:**
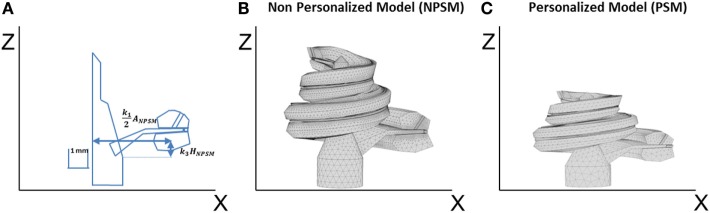
**(A)** Single scaling of the general 3D CAD model of the cochlea based on the *A*, *B*, and *H* values obtained from CBCT preoperative scans. *A* = *k*_1_*A*_NPSM_, *B* = *k*_2_*B*_NPSM_, and *H* = *k*_3_*H*_NPSM_, where *A*_NPSM_, *B*_NPSM_, and *H*_NPSM_ are the non-personalized cochlear features and have values of 10.083, 7.679, and 7.427 mm, respectively. *k*_1_, *k*_2_, and *k*_3_ are factors calculated to obtain the measured *A*, *B*, and *H* values. *k*_2_ is defined in a similar way as *k*_1_ but in the YZ plane; **(B)** generated mesh for the NPSM model; **(C)** generated mesh for the personalized model (PSM) model for patient P1 (*A* = 9.61, *B* = 7.00, and *H* = 2.94 mm).

Finally, the cochlea spiral duct was created using the “Loft” feature of Inventor following a cubic-spline interpolation. Figure 3C presents the spline through the scala tympani used to create the 3D geometry. As a result, we obtained the cochlea mesh presented in Figure 4B.

The described geometry has been used as baseline to create personalized models and for this reason has been termed the non-patient-specific model (NPSM). One of the advantages of the NPSM model is its simplicity, which allows for an easy adaptation to each patient’s specific anatomy by means of scaling. Each compartment was parameterized using dimensions relative to *A*, *B*, and *H*. Particularly, *A* = *k*_1_*A*_NPSM_, *B* = *k*_2_*B*_NPSM_, and *H* = *k*_3_*H*_NPSM_, where *A*_NPSM_ = 10.083 mm, *B*_NPSM_ = 7.679 mm, and *H*_NPSM_ = 7.427 mm are the non-personalized cochlear features, and *k*_1_, *k*_2_, and *k*_3_ are factors calculated to match the measured *A*, *B*, and *H* values.

For example, Figure 4A shows the parameterization at σ = 0°, and Figure 4C presents a scaled cochlea adapted to CBCT measures before implantation using *A* = 9.61, *B* = 7.00, and *H* = 5.94 mm.

### Electrode Array Geometry

Electrode positions were obtained from postoperative CBCT scans. First, the estimates were placed in the geometrical model of the cochlea using generalized orthogonal procrustes analysis (GPA). This method finds the shape correspondence between the preoperative and the postoperative marker points as well as between the preoperative and the CAD marker points presented in Figures 3B,C. GPA estimates a 3D transformation (translation, rotation, and scaling) derived from a “goodness-of-fit” based on the sum of squared errors minimizing the dissimilarity between the preoperative and postoperative marker points. The same method is used to calculate the 3D transformation between the preoperative and CAD marker points. The electrode position estimates from the preoperative scans was converted into the postoperative and the CAD coordinate system using the corresponding GPA transforms.

**Figure 5:**
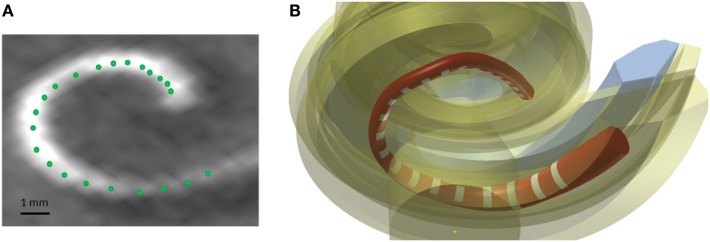
**(A)** Postoperative CBCT scan with the electrode positions. **(B)** CAD model of a Nucleus contour advance electrode array. The red color represents the silicon electrode carrier, the white color represents the electrode contacts, and the yellow color represents the different structures of the cochlea.

A method was developed to improve the estimates of the electrode positions. The estimate is challenging due to the artifacts present in the postoperative scans and the relatively low resolution of the CBCT scanner (voxel size: 0.3 mm × 0.3 mm × 0.3 mm) in comparison to an average electrode spacing of less than around 1 mm. First, the estimates were smoothed fitting a spline through the 22 electrode positions in the CAD coordinate system. Second, the most apical electrode was used as the reference position, and all other electrode position estimates were corrected using the known dimension of the CI24RE or the CI422 Slim-Straight electrode array. Figure 5A presents a postoperative CBCT image with the electrode positions estimated using the method described above marked with green color.

The electrode array was modeled in the CAD domain approximating the size and shape of the Nucleus^®^ CI24RE contour CI array or the CI422 Slim-Straight arrays, which consist of 22 platinum half-band electrodes and additional stiffening rings on a flexible silicone carrier. The length of the CI24RE electrode array is 17 mm. The diameter at the basal part was set to 0.8 mm and at the apical part to 0.5 mm. For the CI422, the insertion depth is 20 mm with a diameter of 0.6 mm and 0.3 mm at the basal and apical end, respectively. Figure 5B shows the CAD model of the electrode array inside the cochlea corresponding with the electrode position estimations presented in Figure 5A.

### Finite Element Method

The CAD model generated in Inventor© was imported into COMSOL© (COMSOL Group, Stockholm, Sweden) to generate a tetrahedral mesh using the general physics algorithm. Three different meshes with different levels of refinement were generated for a mesh convergence study. First, a NPSM with an electrode array placed in a standard position was created based on the general cochlea presented in Figure 3. The minimum element sizes were 5 × 10^−4^, 2 × 10^−4^, and 7.5 × 10^−5^ m, and the total numbers of elements were 16,229, 309,670, and 387,451 for each level of refinement.

The geometry was classified into domains, and each domain was assigned a material property in the form of conductivity. The conductivity values were derived from Briaire (2008) and are listed in Table 1. It was assumed that the conductivities of each domain were linear isotropic. The conductivity of the bone was chosen to be 0.02 as proposed by Whiten (2007) instead of 0.156 as proposed by Briaire (2008).

**Table 1:** **Conductivity values for the cochlea structures (Whiten, 2007; Briaire, 2008)**.

Structure	Conductivity S/m
Electrodes	1000
Scala media	1.67
Scala tympani	1.43
Scala vestibuli	1.43
Basilar membrane	0.0625
Reissner membrane	0.00098
Nerve	0.3
Bone	0.02
Silicone	0.099

Finite element method for the given geometry was solved using the COMSOL Multiphysics v5.0 iterative conjugate gradients solver. A more detailed description of the FEM can be found in Nogueira et al. (2015b). Stationary simulations were performed under the assumption that permittivity effects were negligible. For this reason, the electrical permittivity for all materials was set to 1.

Boundary conditions are modeling constraints required to solve the voltage distribution. Boundary conditions should ideally replicate the physics at the boundary of the modeled domain. This is difficult for simulation of monopolar stimulation because the return electrode lies outside the physical domain of the model. Existing models deal with this issue by assuming that the end of the auditory nerve is grounded, that the ground is infinitely far away, or that the boundary box surfaces are grounded. However, none of these perfectly match the *in vivo* situation (Wong et al., 2016). We simulated monopolar stimulation creating one active electrode within the electrode array in the cochlea and defining the ground as a sphere with a radius of size 50 mm that housed the whole cochlea. The sphere was defined as a bony structure surrounding the cochlea geometry.

It has been shown that placing a ground too close to the cochlea will affect the intra-cochlear current flow in ways that do not match the real-life situation, where the return electrode is relatively far away from the cochlea. As mentioned above, most computational CI models use grounding at infinity, which more or less introduces a potential offset relative to a more realistic approach with a proper return electrode, but which has the benefit of not affecting the intra-cochlear potentials and current flow in the way that an artificial ground placed too close to the cochlea might.

**Figure 6:**
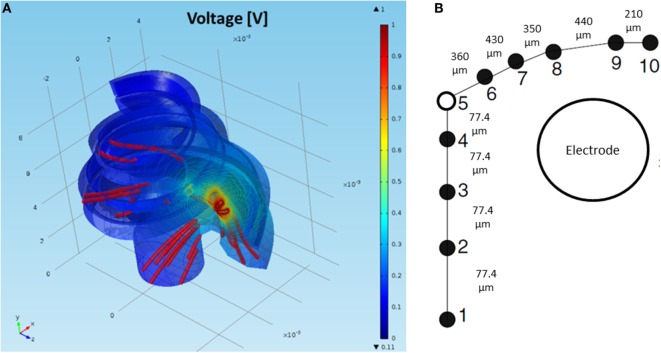
**(A)** FEM simulation of the normalized intra-cochlear voltage for the NPSM model using ground *V*_g3_ when the most basal electrode is stimulated. The colors indicate Volt units. The red lines are current density streamlines. The current exits the cochlea through the modiolus, the basal end of the cochlea, and the cochlea walls. **(B)** Single auditory nerve fiber with *k* = 10 sections.

Figure 6 presents the 3D model simulation for a standard cochlea geometry not adapted specifically to a CI user. Current density streamlines were used to estimate the direction of current flow from the stimulating electrode surface to the return electrode (Tran et al., 2015). The estimated percentage of current passing through the basal end, modiolus, and cochlea walls was 20, 24, and 56%, respectively. These values are in agreement with the simulations performed by Tran et al. (2015).

To measure how the model predictions change as the level of anatomical detail increases, several models were used to simulate the intra-cochlear potentials. The lowest detail model consists of the homogeneous model (HM). This model only takes into account the electrode positions considered as point current sources in an infinite homogeneous medium. The analytical solution for the voltage generated at position (*x*, *y*, *z*) in the medium due to current *I* applied on an electrode *i* at position (*x_i_*, *y_i_*, *z_i_*) is given by:

(1) $$V_{\text{e}}\left(x,y,z\right)=\frac{I}{4\pi \sigma \sqrt{{\left(x_{i}-x\right)}^{2}+{\left(y_{i}-y\right)}^{2}+{\left(z_{i}-z\right)}^{2}}}.$$

This low-level detail model was included in the analysis because it is commonly used in the CI field as a simple model of the electrical field to model further higher level stages of hearing perception (Litvak et al., 2007).

The next level of detail is the NPSM (Figure 4B) followed by a patient-specific model (PSM), which represents the geometry of the patient’s cochlea (Figure 4C). A second version of the NPSM (NPSM2) having averaged dimensions of a set of personalized models was created (Table 3). Finally, an optimized NPSM (optNPSM), an optimized NPSM2 (optNPSM2), and an optimized PSM (optPSM) models were created such that the conductivities were adjusted to measured intra-cochlear potential measurements in CI users. The data collected in CI users are presented in the following sections.

### Intra-Cochlear Potential Measurements in CI Users

Current CI devices are equipped with measurement capabilities that provide enough resolution to capture the intra-cochlear potential at the electrode contacts. An intra-cochlear potential map is built up by consecutive stimulation of each contact from base to apex or *vice versa* (Vanpoucke et al., 2004). Each electrode is stimulated, and the intra-cochlear potential is measured at all contacts, including the stimulating contact. In such a way, a complete potential profile along the scala tympani is obtained. The details of the intra-cochlear potential profiles depend on the anatomy and conductivities of the tissues and on the properties of the electrode contacts. These factors differ from subject to subject and may vary over time (Rattay et al., 2001; Vanpoucke et al., 2004).

Intra-cochlear potentials were measured using the Nucleus Interface Communicator (NIC; Cochlear Corp., Sydney, Australia) to stimulate and record from the electrodes of each Nucleus CI user. Each electrode was stimulated using biphasic pulses (cathodic first). The phase width was set to 25 μs with an 8-μs phase gap. The electrodes were stimulated in monopolar MP2 mode meaning that current flowed between the active intra-cochlear electrode and the plate electrode placed in the implant. The amplitude of the pulses was set to 106.50 μA. The backward telemetry of NIC offered six recording samples per phase. Sample 6, the last sample of the first phase, was used as an estimation of the intra-cochlear voltage for each pair of stimulating and recording electrode. During this manuscript, the recordings at the stimulating electrode will not be considered, as these values are dominated by the electrode–tissue impedance and not by the anatomy. It is worth mentioning that the voltage is recorded simultaneously to the stimulation, and the neural response occurs few milliseconds after stimulation. For this reason, the neural response does not influence the voltage at the electrode positions.

### Validation Measures

The FEM model of the electrically stimulated cochlea was used to simulate intra-cochlear potentials. A validation measure is defined to compare the measured and the modeled intra-cochlear potentials. The intra-cochlear potential root-mean-square error (RMS_error_) is defined as the difference between the measured and the modeled impedance values averaged for all electrodes (Whiten, 2007):

(2) $${\text{RMS}}_{\text{error}}=\sqrt{\frac{1}{N\cdot \left(N-1\right)}\sum_{s=1}^{N-1}\sum_{k\neq s}{\left\{\left({\text{VM}}_{k}^{s}-{\bar{\text{VM}}}^{s}\right)-\left({\text{VP}}_{k}^{s}-{\bar{\text{VP}}}^{s}\right)\right\}}^{2}},$$

where *N* represents the number of active electrodes, and ${\text{VM}}_{k}^{s}$ and ${\text{VP}}_{k}^{s}$ are the measured and predicted voltages at electrode *k* when electrode *s* is stimulated. The mean across *N* − 1 recorded voltages for each electrode *s* being stimulated is denoted by ${\bar{\text{VM}}}^{s}$ and ${\bar{\text{VP}}}^{s}$ for the measured and predicted voltages, respectively. The mean values were subtracted to remove any bias between the measured and the predicted data.

### Model Optimization

**Figure 7:**
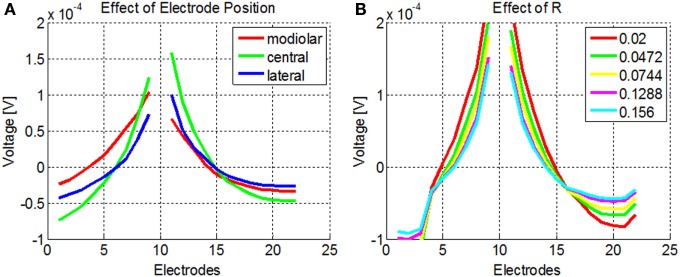
**(A)** Effect of different ratios *R* = σ_ST_/σ_B_ on the intra-cochlear voltage recordings when electrode 11 is stimulated using the NPSM model. With decreasing values of *R*, the voltage distribution becomes wider. The mean of the voltage distribution has been subtracted. **(B)** Effect of different electrode positions on the intra-cochlea voltage distribution. The central positions correspond with the electrode positions estimated from the imaging data, and the modiolar and lateral positions correspond with a displacement of 0.5 mm toward the modiolar or lateral direction. The voltage distribution model predicts wider intra-cochlear voltage distribution when the electrodes are shifted toward the modiolus.

The NPSM and PSM models were designed using fixed parameter values. Given a personalized cochlea geometry and electrode positions, it is possible to optimize the model modifying the parameters such that the difference between the modeled and measured intra-cochlear voltage values is minimized. It has been shown that the ratio between the conductivity values of the scala tympani and the bone *R* = σ_ST_/σ_B_ plays a major role in defining the current paths in the cochlea. For example, keeping the σ_ST_ constant and lowering the σ_B_ to 0.0017 Sm^−1^ has an effect on both the magnitude and shape of the current distribution across cochlear position as shown by Frijns et al. (1995) and Hanekom (2001). This effect is shown in Figure 7A where simulations of the intra-cochlear voltage distribution are presented using the NPSM model for different ratios *R* when electrode 11 is stimulated. The value of σ_ST_ was kept constant, and the value of σ_B_ was varied resulting in the different ratios *R*.

The PSM model relies on the quality of the electrode position and cochlear extent estimation to provide accurate estimations of the intra-cochlear potentials. Errors in the electrode position have effects on the shape of the current distributions. As an example, Figure 7B shows the intra-cochlear voltage distributions for a PSM model using the electrode positions estimated from CBCT data (central), shifting all electrode positions by 0.5 mm toward the modiolus (modiolar) and toward the lateral wall (lateral). It can be observed that the model predicts wider intra-cochlear voltage distributions when the electrodes are placed closer to the modiolus.

Given that the ratio *R* is not known for each CI user and that the electrode position estimation may contain errors, we decided to optimize the conductivity value of the bone individually. The optimization was based on finding the parameter *R* that minimized the RMS_error_ for each CI user, i.e.,

(3) $$R_{\text{min}}=\underset{R}{\text{argmin}}\left({\text{RMS}}_{\text{error}}\right).$$

The *R*_min_ parameters will optimize the model given the geometry and electrode positions estimated for each CI user.

### Auditory Nerve Model

**Figure 8:**
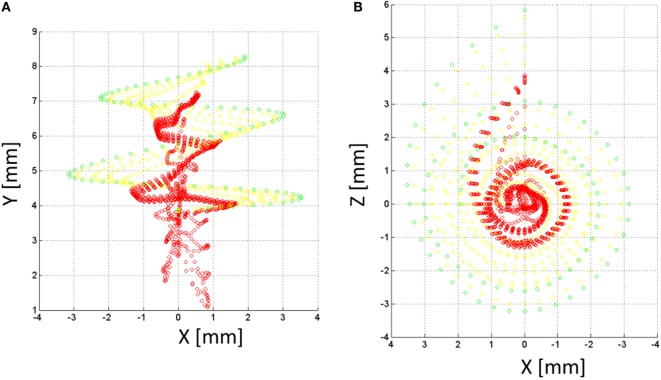
**Nerve fibers on the XY (A) and XZ (B) planes for the NPSM model**. In total, 9000 nerve fibers were modeled. For better representation, only 900 fibers are shown in the figure. Each fiber is composed by 10 nodes. The most peripheral node (node 10) is represented in green color, nodes 9 and 8 are represented in yellow color, and the rest of nodes are represented in red color.

A model of the auditory nerve activity was coupled to the voltage distribution model. The model is based on the model of Litvak et al. (2007), and it assumes the following: (1) there exist a finite number of discrete neuronal elements spread out over the cochlear space, (2) these elements have a range of thresholds drawn from a log-normal distribution, (3) the electric field at a given spatial location is obtained from a FEM simulation. The physiology of the auditory nerve fiber was modeled based on a simplified version of Smit et al. (2008). Each nerve fiber is composed by *k* = 10 sections, nodes 10–6 corresponded to the dendrites, node 5 to the soma, and nodes 4–1 to the axon (Figure 6B). In total, 7000 nerve fibers all along the cochlea were simulated. Figure 8 shows the distribution of the nerve nodes along the cochlea for the NPSM model. The same scaling method used to create the PSM models was used to scale the nerve fibers. The internode distance, however, was kept the same for all models. The voltage distribution is sampled in each nerve section and is denoted as *V_i_(k)*, where *k* denotes the section, and *i* denotes the nerve fiber. For each nerve fiber, the activation function is computed discretizing the second derivative of the voltage distribution along the nerve axon (Eq. 4). The assumption is that for long homogeneous fiber (i.e., an unmyelinated axon), the neural elements are cylinders of constant diameter and length (Rattay, 1999; Rattay et al., 2001).

(4) $$D_{i}\left(k\right)=\frac{V_{i}\left(k-1\right)-2V_{i}\left(k\right)+V_{i}\left(k+1\right)}{\Delta x^{2}},$$

where Δ*x* denotes the length of the neural elements.

The computational model of each node of the auditory nerve model is very similar to the one presented by Litvak et al. (2007). The spike timing is neglected, and the spike count is summed for each time frame. To compute the number of neurons firing *N*(*x*), each neuron is modeled independently. For a neuron *i* at position *X*_i_ (*k*), the firing probability is equal to:

$$P\left(k\right)=\emptyset \left[\frac{\left|A_{i}\left(k\right)\right|-A_{{\text{thr}}_{\text{i}}}\left(k\right)}{A_{\text{thr}}\left(k\right)\cdot \text{RS}\left(k\right)}\right],$$

where ∅ is the cumulative normal distribution function*, A*_thr_ (*k*) is the electrical activation required to reach the neuron’s threshold, and RS is the neuron’s relative spread (Bruce et al., 1999).

For the simulations, thresholds *A*_thr_ (*k*) were assigned randomly from a log-normal distribution with the ratio of SD to mean set to 0.3 (Litvak et al., 2007). For computational convenience, the mean of the threshold distributions was arbitrarily set at 0 dB relative to units of *A_i_* (*k*). As in Litvak et al. (2007), the RS of each neuron was chosen from a normal distribution with a mean of 0.0635 and an SD of 0.04. The RS was not allowed to go below 0.03 or above 0.10. For simplicity, only the activation function for the first node (*k* = 10) was used. The model was applied to compute the summed neuronal activity ∑N(x) under monopolar stimulation for each electrode.

## Results

### Subjects

Twelve adult CI users participated in the study. For each CI user, preoperative and postoperative imaging data were extracted to characterize their cochlear extent and electrode positions. Table 2 presents the subject details. CBCT imaging was acquired in clinical routine. Therefore, there was no additional radiation to the patients. After explanation of the study protocol and the risks and benefits of participating, all subjects signed a consent form before participating. The study protocol was approved by the institutional medical ethics committee, and all subjects gave their informed consent to participate in the study.

**Table 2:** **Subject details**.

ID	Age (years)	Duration of deafness (years)	Cause of deafness	Implant experience in years	Electrode type	Stimulation rate (pulses/s)
P1	48	0	Trauma	2.8	RE-24CA (left)	900
P2	44	0.42	Sudden hearing loss	5	CI512 (left)	900
P3	44	22.92	Unknown	2.5	CI512 (left)	900
P4	78	8.92	Sudden hearing loss	1.8	CI24RE (right)	900
P5	65	0	Sudden hearing loss	1	RE-24CA (left)	900
P6	68	9.59	Unknown	4.2	RE-24CA (left)	900
P7	51	0	Sudden hearing loss	3.2	CI422 (right)	900
P8	23	19.42	Neonatal jaundice	3.9	CI512 (right)	900
P9	73	11.92	Unknown	4.2	CI512 (left)	900
P10	71	0.00	Unknown	3.7	CI512 (right)	900
P11	57	0	Sudden hearing loss	3.8	CI512 (right)	900
P12	55	0	Sudden hearing loss	1.5	CI24RE (right)	900

### CBCT Measurements

Imaging of the temporal bone is challenging due to its small size and high bone density. We consulted one expert to measure the *A*, *B*, and *H* values as well as the electrode positions. Table 3 shows the averaged *A*, *B*, and *H* values for the 12 CI users participating in the study.

**Table 3:** ***A*, *B*, and *H* values for each CI user in (mm)**.

ID	*A (mm)*	*B (mm)*	*H (mm)*
P1	9.61	7.00	4.94
P2	7.81	5.49	5.27
P3	9.72	6.85	5.13
P4	8.91	6.59	4.77
P5	9.00	6.80	4.76
P6	9.85	7.47	4.01
P7	8.91	6.53	4.50
P8	8.75	6.95	5.01
P9	8.79	6.28	4.805
P10	8.88	6.68	4.94
P11	7.50	5.70	4.32
P12	8.98	6.95	4.99
Mean	8.89	6.61	4.78

### Voltage Distribution Simulations for Individual CI Users

**Figure 9:**
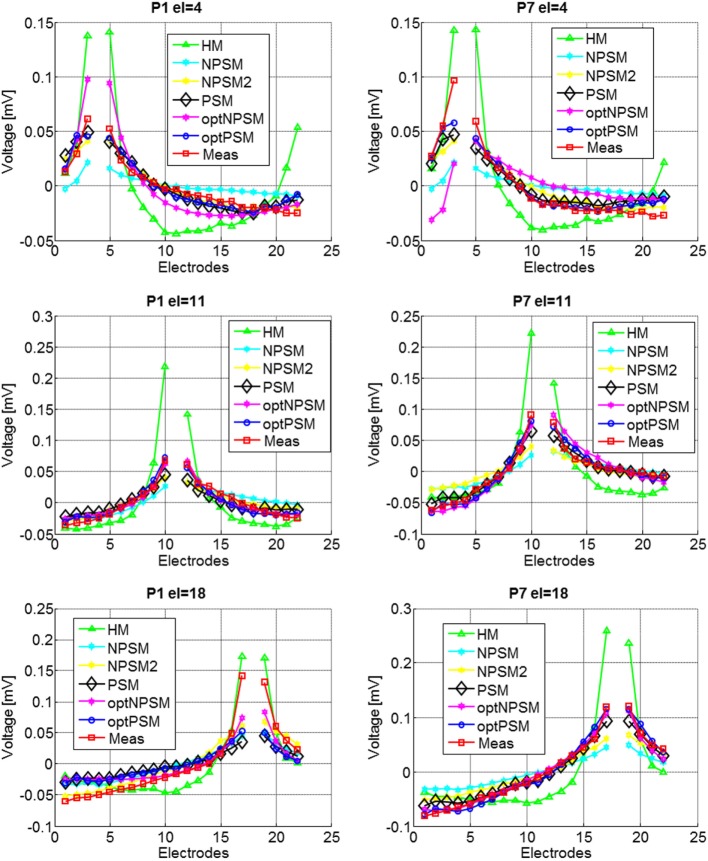
**Voltage distribution measurements for two CI users, P1 (left panel) and P7 (right panel) participating in the study for stimulating electrodes 4 (top panel), 11 (central panel), and 18 (bottom panel)**. The mean across electrodes has been subtracted from each voltage distribution.

Figure 9 presents the experimental measurement ${\bar{\text{VP}}}^{s}$ and the model measurements ${\bar{\text{VM}}}^{s}$ with different levels of detail (HM, NPSM, NPSM2, PSM, optPSM, and optNPSM) for CI users P1 and P7 at three stimulating electrodes *s* = 4, 11, and 18.

### Model Results

**Figure 10:**
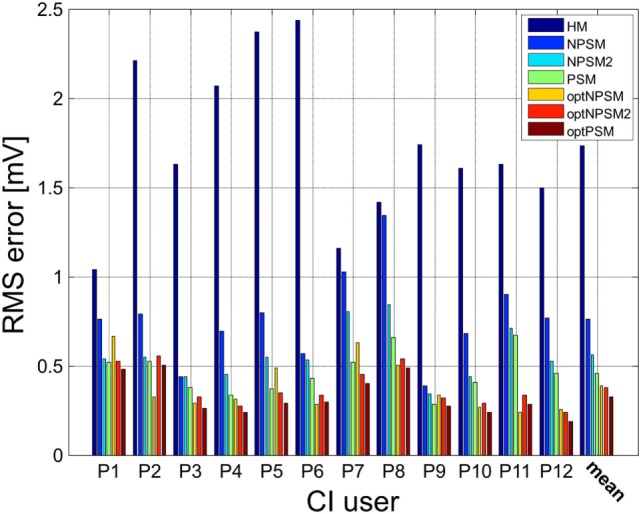
**Patient-specific and averaged RMS_error_ between measured and modeled intra-cochlear potentials for each CI user for the HM, NPSM, NPSM2, PSM, optNPSM, optNPSM2, and optPSM models**. With increased level of detail the RMS_error_ is reduced. Moreover, it can be observed that the RMS_error_ can be minimized by adapting the *R* ratio.

For each electrode, we computed the RMS difference between the measured and the modeled impedance values using Eq. 2. The RMS_error_ was computed for the HM, NPSM, NPSM2, and PSM models. Figure 10 presents the results averaged for all stimulating electrodes for the 12 CI users participating in the study.

The resulting data were compared using one-way repeated measures of analysis of variance (ANOVA) to test for the effect of model. If a statistically significant effect was found, pairwise comparisons were made using two-tailed *t*-tests with Bonferroni corrected *p*-values. Also, 15 pairs of data models were compared, and therefore a correction factor of 15 was applied to all *p*-values.

A significant effect of model was observed [*F*(1.398, 15.373) = 59.925, *p* < 0.0005]. The results show that the RMS_error_ decreased with each level of refinement. The HM model produced the largest error (1.7 mV) followed by the NPSM (0.76 mV), the NPSM2 (0.56 mV), and the PSM models (0.46 mV). Note that the HM model obtained the largest RMS_error_ although the conductivity value for this model was selected such that it minimized the average error for all electrodes and CI users with respect to the measured data. The NPSM2 model significantly reduced the RMS_error_ with respect to the NPSM model, meaning that adapting the geometry of the cochlea to patient data in a general way improves the voltage distribution prediction. The PSM significantly reduced the RMS_error_ in comparison to the HM, the NPSM, and the NPSM2 models. The results here presented demonstrate that personalizing the geometry and the electrode positions based on imaging data can improve the intra-cochlear potential predictions at the electrode locations.

### Example of Parameter Optimization

Equation 3 was used to optimize the PSM, NPSM, and NPSM2 models. The voltage distribution was simulated for 20 values of *R* in the interval [9.16, 71.5] using the NSPM, the NPSM2, and the PSM model for each CI user. From the 20 solutions, the *R* delivering the minimum RMS_error_ was selected as the optimum model. The mean *R*_min_ for the optPSM, optNPSM, and optNPSM2 models were 54.44, 46.54, and 18.73, respectively. Table 4 presents the optimal *R*_min_ for each CI user using the PSM, NPSM, and NPSM2 models, respectively. From these tables, it can be observed that the NPSM requires larger adjustments in the *R* ratio than the NPSM2 and the PSM models to be fitted to the measured data. For the PSM model, it seems that an *R* value corresponding to a bone conductivity close to 0.02 Sm^−1^ can be used to approximate the measured data. As expected, Table 4 shows that using a more realistic geometry such as the NPSM2 model requires smaller adjustments in the *R* ratio than using a more unrealistic geometry such as the NPSM model.

**Table 4:** ***R*_min_ values obtained after optimizing the PSM, the NPSM, and the NPSM2 models for each CI user**.

ID	*R_min_ NPSM*	*R_min_ NPSM2*	*R_min_ PSM*
P1	30.97	62.14	68.38
P2	11.10	46.56	65.26
P3	18.51	52.79	71.50
P4	15.39	30.30	30.30
P5	30.3	71.50	71.50
P6	9.16	40.33	59.03
P7	19.22	59.03	55.91
P8	30.30	71.50	71.50
P9	12.27	30.30	30.30
P10	9.17	22.74	30.30
P11	19.22	43.44	65.26
P12	19.22	27.86	30.97
Mean	18.73	46.54	54.44

*The *R* values have no units as it is obtained after division of two conductivity values*.

The results presented in Figure 9 show that optimizing the NPSM (optNPSM), the NPSM2 (optNPSM2), and the PSM (optPSM) models significantly reduced the RMS_error_ to 0.38, 0.38, and 0.32 mV, respectively. It is remarkable that the optPSM provided a small significant improvement with respect to the PSM model. The optNPSM model produced a large significant reduction in RMS_error_ with respect to the NPSM model. No significant differences could be observed between any combination of the optNPSM, the optNPSM2, and the optPSM models or between the optNPSM, the optNPSM2, and the PSM models.

### Analysis of the Activation Function

**Figure 11:**
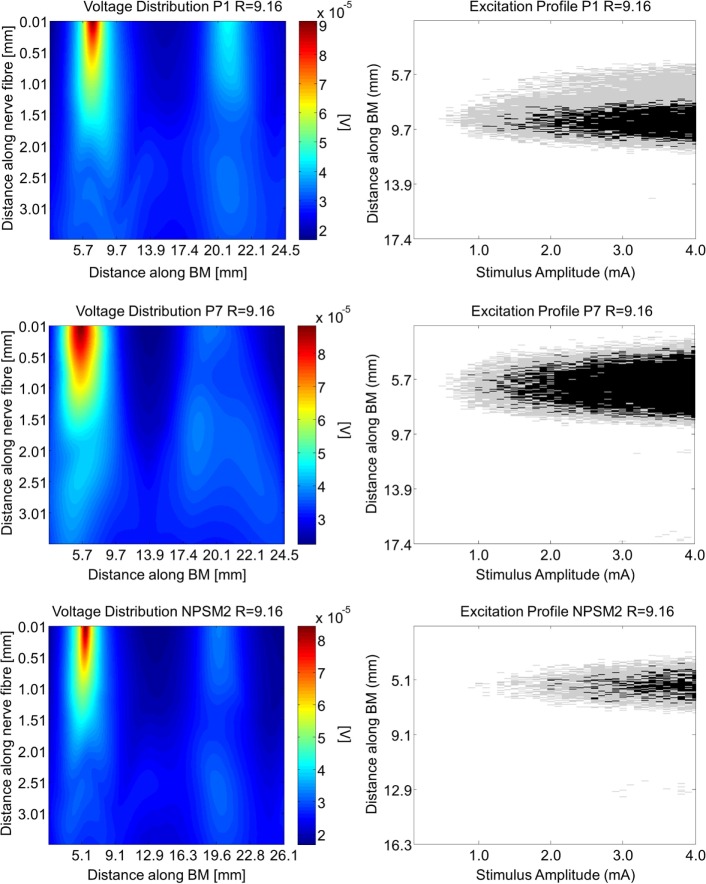
**Voltage distribution, activation function, and excitation profiles when electrode 10 is stimulated using the P1, P7, and the NPSM2 models with a conductivity ratio *R* = 9.16**. The left panel shows the voltage distributions in volts [V] along each nerve fiber and along the basilar membrane. The central panel presents the corresponding activation function in [V/mm^2^]. The right panel indicates which neurons are excited when stimulating electrode 10 (excitation profile). The black areas in the excitation profile indicate stimulation in the axon, the gray areas represent stimulation in the most peripheral process, and the white area means no excitation.

**Figure 12:**
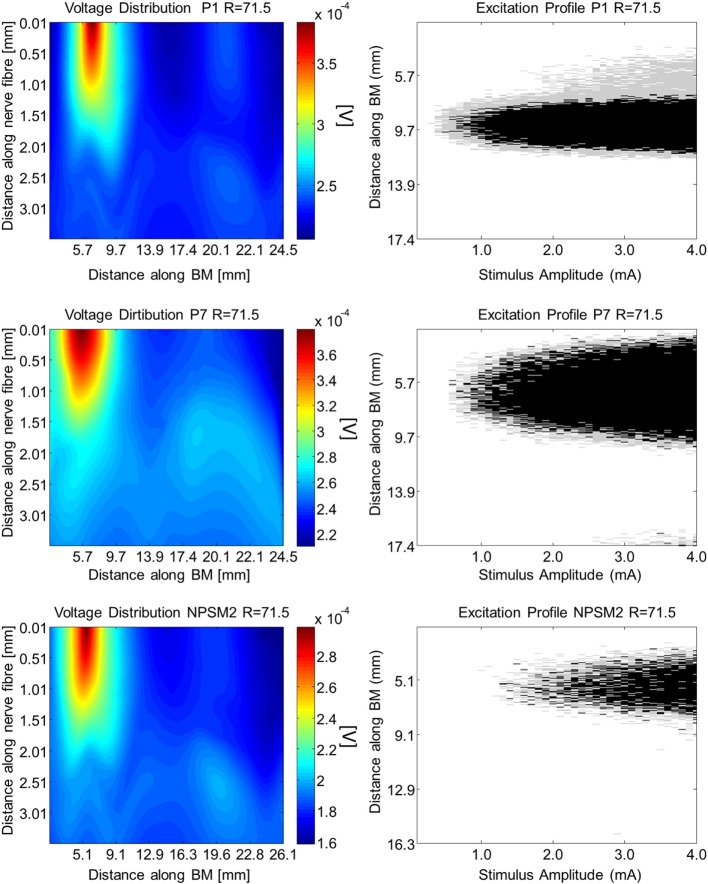
**Voltage distribution, activation function, and excitation profiles when electrode 10 is stimulated using the P1, P7, and the NPSM2 models with a conductivity ratio *R* = 71.5**. The left panel shows the voltage distributions in volts [V] along each nerve fiber and along the basilar membrane. The central panel presents the corresponding activation function in [V/mm^2^]. The right panel indicates which neurons are excited when stimulating electrode 10 (excitation profile). The black areas in the excitation profile indicate stimulation in the axon, the gray areas represent stimulation in the most peripheral process, and the white area means no excitation.

Using the activation function provided in Eq. 4 and the auditory model presented in Section “Auditory Nerve Model,” we give insight on the effects of different conductivity values as well as geometries in the activation function and auditory nerve excitation profiles. Figures 11 and 12 present the voltage distribution, the magnitude of the activation function, and the excitation profile for the NPSM2, and two versions of the PSM models (P1 and P7) when electrode 10 is stimulated. Excitation profiles (Kalkman et al., 2014) indicate which neurons are excited when stimulating a given electrode. The activation function given in Eq. 4 was estimated in 10 μm length segments. The black areas in the excitation profile indicate stimulation in the soma (node 5), the gray areas represent stimulation in the peripheral process (node 1), and the white area means no excitation.

From Figures 11 and 12 it can be observed that increasing the conductivity of the bone (i.e., decreasing the ratio *R*) in any of the models reduces and narrows down the magnitude of the voltage distribution and the excitation profiles. Figures 11 and 12 show large variability in the voltage distributions and excitation profiles for the different models. For example, the peak magnitude, peak location, and width of the voltage distribution change considerably from model to model. This variability is not only caused by the dimensions of the cochleae and the electrode placements but also by the different relative positions between the electrodes and the nerve fibers. Given that the models have different cochlear geometries and electrode placements, it is difficult to compare the prediction of the activation functions. Instead, we estimated the peak position and the width of the voltage distribution at the auditory nerve positions for the most peripheral along the basilar membrane. We analyzed the peak position and the width of the voltage distribution when electrode 10 was activated for the different PSM models. Note that the voltage distribution will be influenced by different cochlear sizes and different insertion angles when electrode 10 is stimulated. In this study, an SD in peak location of 2.5 mm for the peripheral node across the 12 PSMs was observed. The SD of the 3 dB bandwidth in the voltage distribution at the nerve positions across the 12 PSMs was 3.0 mm. These values are in the range of values reported by Malherbe et al. (2015a,b). The SD in 3 dB bandwidth for the NPSM2 model for different values of *R* (from 9.16 to 71.5) was 8.1 mm, which is much larger than the variability observed across PSMs. However, the peak location did not change for different values of *R*. Therefore, from this study, it can be concluded that the procedure to optimize the *R* ratio based on intra-cochlear potentials will also change the estimation of voltage spread at the level of the auditory nerve positions, but not the peak location of the excitation using a non-personalized model. It still remains a question whether the optimization at the level of the intra-cochlear voltage distribution results in more realistic simulations of the activation function and the excitation profiles. Obviously, the peak location cannot be optimized using this optimization; however, it is possible that the width of the voltage distribution becomes more realistic.

## Discussion

Within this manuscript, a methodology to construct a patient-specific 3D volume conduction model of the cochlea based on clinical CBCT imaging data is presented. Volume conduction models are necessary to simulate the voltage at the positions of the auditory nerve and simulate their activity (Smit et al., 2008). A first step to develop volume conduction models is to predict the intra-cochlear voltage distribution at the electrode positions. Commercial CIs can be used to measure the intra-cochlear voltage distributions and therefore, the models can be validated.

The model presented in this manuscript is simple and allows a flexible adaptation to each individual’s cochlea and electrode positions. For example, the model of Malherbe et al. (2015a,b) is constructed using greater detail including the height of the ducts, the width of the bony canal in which the cochlear nerve lies, the inclusion of a head model, and the consideration of the reference electrode. The simpler and less accurate geometry used in this study can be adapted to clinical CT scans using the electrode positions and just six parameters to characterize the cochlea. However, the model will not take into account the effects of having a realistic head, some details of the cochlear ducts, and the position of the reference electrode. For this reason, a correction of the model predictions is required. In this study, we suggest to correct the model predictions adapting the electrical parameters to measured voltage distributions in CI users. This adaptation requires an individualization process. First, imaging data are extracted pre- and post-cochlear implantation. Second, a generalized CAD cochlea model is created such that the parameters from the imaging data can be used to adapt it to each CI user. Third, the electrodes are placed in the CAD cochlea model according to CBCT measurements. Fourth, FEM is used to estimate the intra-cochlear voltage distributions. The RMS_error_ between the measured and predicted intra-cochlear voltages was used to assess the quality of the predictions. Four models were compared, a HM that only takes into account the electrode positions, two versions of a non-patient-specific model (NPSM and NPSM2), and a patient-specific model (PSM). Results show that the PSM significantly reduces the RMS_error_ by 1.3, 0.3, and 0.1 mV with respect to the HM, NPSM, and NPSM2 models, respectively. An algorithm has been proposed to optimize the ratio between the conductivities of the scala tympani and the bone such that the RMS_error_ is minimized. Using this optimization in the NPSM model (optNPSM) produces a large and significant reduction in RMS_error_ similar to the PSM model. Therefore, if imaging data for a particular CI user are not available, the optNPSM is proposed as the best model to simulate intra-cochlear potential distribution. Optimizing the PSM model (optPSM) such that the RMS_error_ is minimized constrained to the patient-specific geometry produces a small but significant RMS_error_ reduction of 0.14 mV with respect to the PSM model. Therefore, the optPSM is the most accurate model to simulate the intra-cochlear potentials at the electrode locations.

Comparing the voltage distribution at the nerve fiber positions between different geometries is challenging. The cochlear geometry, the electrode positions, and the nerve fiber positions contribute to large differences between the models. A large cochlea such as the NPSM model shows wide activation functions in comparison to smaller cochleae such as the PSM models. In all models, it is shown that the conductivity of the bone can be used to reduce the magnitude and width of the activation function. Therefore, adapting the NPSM model to impedance data of each CI user may be useful to approximate the real activation function.

Malherbe et al. (2015a,b) modeled potential distributions and neural excitation profiles (threshold amplitudes, center frequencies, and bandwidths) for different user-specific cochlear morphologies and electrode placements within the cochlea. In general, they showed that the variability of threshold, characteristic frequency, and bandwidth values observed as a result of morphology are almost as large as variations observed as a result of electrode placement, suggesting that user-specific morphology is an important determinant of CI performance. For example, they showed a maximum difference of around 2.1 mm in characteristic frequency for different cochlear morphologies. In this study, an SD of 2.5 mm in peak location was observed across different PSMs, i.e., morphologies and insertion angles. Malherbe et al. (2015a,b) showed that the threshold width had a maximum difference of 1.9 mm at 3 dB bandwidth. In our study, an SD of 3.0 mm in 3 dB bandwidth of the voltage distribution was observed.

It needs to be remarked that the absolute RMS_error_ of the intra-cochlear voltage distribution obtained with the optPSM model is still relatively high. There are at least three reasons that contribute to the error of the model predictions. (1) Errors in the estimation of the electrode positions and characterization of each individual’s cochlea; (2) the fact that tissue growth around the electrodes is not modeled; (3) correct modeling of the model boundary conditions which in turn influence the current paths in the cochlea; (4) the absence of structures surrounding the cochlea or a realistic human head.

### Measurements from CBCT Data

Imaging of the temporal bone is challenging due to its small dimensions and its high bone density. Moreover, the artifacts produced by the electrode contacts limit the accuracy of the electrode position determination. The resolution of the CBCT scan used in this manuscript was 0.3 mm × 0.3 mm × 0.3 mm, which is probably not sufficient to obtain accurate measurements of electrodes spaced by less than 1 mm. In order to improve the estimation of the electrode positions, we first fitted a curve through all the estimated positions and second, the measured data was corrected using the known dimensions of the Nucleus electrode arrays. Still, the use of newer imaging techniques (Pearl et al., 2014a,b) with higher resolution will allow the improvement of electrode position estimation potentially reducing the error predictions of the model.

### Non-Modeled Tissue Growth

Tissue growth around the electrodes has been observed in CIs (Huang et al., 2007). Tissue growth will be dependent on each CI user and also on each electrode. In Hanekom (2005), an electric and neural model of a CI with and without tissue encapsulating the electrodes was presented. Significant different results were found for electrodes with or without taking the encapsulation into account, suggesting that electrode encapsulation can alter threshold currents and spread of excitation. In our model, we did not simulate the potential tissue growth around electrodes. If there would be a method to estimate *in vivo* the amount of tissue growth, this could be incorporated into the model to improve the model predictions.

### Current Paths in the Cochlea

It has been shown that the current exits the cochlea through the modiolus (14%), the basal end (22%), and through the cochlea walls (64%) (Tran et al., 2015). These estimations were obtained from a complex FEM model including a model of the CI user’s head. These percentages are general and may vary on each CI user depending on the morphology of the internal auditory system. The model presented throughout this manuscript is based on a generalized model (NPSM) that uses an infinite ground. Using this model, the current paths estimated are modiolus (16.01%), basal end (30.08%), and cochlear walls (53.91%), which are in the range of the published data by Tran et al. (2015). Furthermore, we presented an optimization method to adapt the ratio between the conductivities of the scala tympani and the bone for each CI user using a much simpler FEM model without modeling a patient’s head. This optimization will modify the current paths in the cochlea at the expense of improving the intra-cochlear voltage distribution.

### Structures Surrounding the Cochlea

It has been shown that the morphology of the structures surrounding the cochlea and the location of the extra-cochlear electrodes determine the path that current will follow through the cochlea. Head models have been introduced into the FEM simulations to investigate how the current paths in the cochlea are affected. These studies have found that neural excitation patterns are affected by the implementation of the return electrode and structures surrounding the cochlea (Malherbe et al., 2015a,b; Tran et al., 2015).

### Applications of the Model and Future Work

The model has potential to be applied to create new sound coding strategies. For example, FEM models are used to analyze simultaneous stimulation sound coding strategies (Kalkman et al., 2014, 2016). These strategies try to shape or focus the electrical field to be more focused and to produce different pitch sensations (Nogueira et al., 2009, 2015a). Patient-specific 3D volume conduction models can be used to optimize the parameters of the focusing and the current steering coefficients (Litvak et al., 2007).

Another potential application is the prediction of higher level perceptual mechanisms. One critical aspect in the rehabilitation of hearing with CIs is the fitting of the device. Here, the minimum levels and most comfortable levels need to be estimated for each CI user. The device fitting is time consuming due to a large iterative subjective procedure. The fitting levels (loudness perception) depend not only on the current spread in the cochlea but also on the auditory nerve activity. As shown in this manuscript, the voltage predicted by the volume conduction model can be sampled at the auditory nerve positions and coupled to an auditory nerve model (Smit et al., 2008; Nogueira et al., 2015a,b). Such a model could be used to predict the loudness of sound and is a potential tool to speed up and objectivize the fitting procedure.

## Author Contributions

WN designed the geometrical and computational model; designed experiments; designed the measurement system to record voltage distribution; and wrote the manuscript. DS contributed to the design of the geometrical model and contributed to manuscript writing. AB contributed to manuscript preparation. RP contributed to imaging data collection and contributed to manuscript preparation. WW contributed to the geometrical model design; contributed to the imaging data collection; and contributed to manuscript preparation.

## Conflict of Interest Statement

The authors declare that the research was conducted in the absence of any commercial or financial relationships that could be construed as a potential conflict of interest. The reviewer EY and handling Editor declared their shared affiliation, and the handling Editor states that the process nevertheless met the standards of a fair and objective review.
